# Pasta Enriched with Dried and Powdered Leek: Physicochemical Properties and Changes during Cooking

**DOI:** 10.3390/molecules27144495

**Published:** 2022-07-14

**Authors:** Beata Biernacka, Dariusz Dziki, Urszula Gawlik-Dziki

**Affiliations:** 1 Department of Thermal Technology and Food Process Engineering, University of Life Sciences in Lublin, 31 Głęboka St, 20-612 Lublin, Poland; beata.biernacka@up.lublin.pl; 2 Department of Biochemistry and Food Chemistry, University of Life Sciences in Lublin, 8 Skromna St, 20-704 Lublin, Poland; urszula.gawlik@up.lublin.pl

**Keywords:** pasta, leek, cooking properties, mechanical properties, antioxidant activity, quality

## Abstract

Food enrichment is commonly applied to increase the pro-health and dietary value of final products. This study aimed to evaluate how green leek powder (GL) added to semolina influenced the physicochemical, sensory, and cooking characteristics of the pasta. The pasta was prepared by partially substituting semolina with 0, 1, 2, 3, 4, and 5 g/100 g of GL. Then, the pasta samples were assessed for color, cooking properties, sensory features, mechanical properties, total phenolic content (TPC), and antioxidant activity (AA). The results indicated that GL significantly decreased the lightness and increased the yellowness of cooked pasta. The total color difference between the control pasta and enriched pasta samples ranged from 2.54 to 6.89 and 5.60 to 11.06 (for uncooked and cooked pasta, respectively). The addition of GL also caused an increase in the optimal cooking time and cooking loss. Moreover, a significant increase in stretching force was observed in cooked pasta. Sensory evaluation revealed that substitution of semolina with up to 2 g/100 g GL did not affect the smell, taste, and color of pasta. Most importantly, GL-enriched pasta was characterized by higher TPC and higher AA compared to the control samples. In summary, our results recommend partial replacement of semolina by GL (up to 3 g/100 g) in pasta production.

## 1. Introduction

Wheat pasta (WP) is one of the most popular cereal products due to its low production cost, high energy value, ease of preparation, and long shelf life [1]. WP is available in different varieties with varied compositions of ingredients, quality features, dimensions, shapes, and additives [2]. Traditionally, WP has been produced from durum wheat (*Triticum durum*) using a procedure that involves mixing semolina with water, dough extrusion or lamination, and drying [3]. WP is characterized by excellent cooking and sensory properties and is also rich in easily digestible carbohydrates and protein, as well as a low amount of fiber, vitamins, and antioxidants. It is well known that these nutrients play a crucial role in the prevention of many diseases such as cancer, diabetes, and cardiovascular disorders [4,5].

The composition of the raw materials used for pasta production can directly influence the physical, chemical, and textural properties of the final product [2]. Recent studies have focused on the enrichment of WP [6] with the aim of improving its functional properties. The additives used for enrichment include not only vegetables and fruits [7,8] but also unconventional ingredients, such as vitamins [9], insects [10], minerals [11], and natural dyes [8,12].

Dried and powdered leek can be an interesting additive for WP. Leek (*Allium porum* L. or *Allium ampeloprasum* var. *porum*) is a vegetable commonly used in European countries ranging from the Balkan Peninsula to Ireland and Western Asia. Similar to garlic and onions, it belongs to the family of *Liliaceae* and the genus *Allium*. Leek is used for processing, medicinal, and culinary purposes [13]. Leeks, as well as other allium vegetables, are rich in secondary metabolites, including phenolic acids and their derivatives and flavonoids. Studies have shown that the major flavonoid aglycone is kaempferol [13], which is abundant in leeks. Fresh leek contains a large amount of carbohydrates, protein, and digestive fiber. It is also a good source of nitrates and various sulfonic acids and hence imparts a rich taste [14]. Moreover, the leek is rich in nutrients, including vitamins C, B_6_, A, and E, niacin, folic acid, and minerals such as calcium, iron, potassium, magnesium, sodium, copper, phosphorus, zinc, and manganese [15]. Due to the high content of nutrients, the leek is included in weight loss diets [16]. In addition, the leek has secondary metabolites with anti-inflammatory and antioxidant activities and can therefore be used as an alternative to anti-inflammatory agents in the treatment of conditions such as allergies [17]. It is well known that natural and clean essential oils should be used in food [18]. Leek essential oil is rich in several valuable compounds, mainly allyl sulfide [16].

In industrial production, additives such as spinach, tomatoes and pumpkin are often used to fortify pasta. Recent works in this field also concern the possibility of adding vegetables such as carrots, beetroot or celery [6]. In an earlier study, semolina flour was also replaced with onion peel powder at various concentrations, and the changes in the nutritional, technological, and sensory properties of pasta were examined [19]. The enrichment of wheat pasta with vegetables enhances its nutritional and pro-health attributes. On the other hand, such modifications often have a negative influence on pasta cooking quality and sensory acceptance. Thus, the level of the individual ingredients has to be precisely estimated before their incorporation into pasta. A recent study [20] proved that the green parts of leek contain a higher amount of ash and fiber compared to the white parts. Moreover, green leek powder (GL) is a valuable source of biologically active compounds, especially phenolic acids, of which ferulic acid is dominant [20]. Dried leek can be added to soups, sauces, salads, casseroles, and meat dishes and is also a good alternative to fresh vegetables. Leek can improve the taste of a dish, imparting slight spiciness. Previous studies have also shown that it can be used as an additive in the production of bread [21], traditional Greek sausages [22], and fermented sausages [23]. However, the physicochemical properties of pasta prepared from semolina partially substituted by dried leek have not been studied yet. The present study aimed to evaluate the quality and antioxidant properties of durum WP enriched with dried and powdered green parts of the leek.

## 2. Results and Discussion

### 2.1. Basic Composition of Raw Materials and Pasta

Table 1 presents the chemical composition and water activity (a_w_) of control pasta and pasta enriched with GL at concentrations of 0, 1, 2, 3, 4, and 5 g/100 g (PL0, PL1, PL2, PL3, PL4, and PL5, respectively).

Enrichment of pasta with GL resulted in a slight but significant decrease in protein content but an increase in ash, fiber, and fat content. A previous study proved that GL contains higher amounts of ash and fiber compared to white leek parts [20]. The water activity (a_w_) of pasta samples ranged from 0.537 to 0.570 (PL0 and PL5, respectively). A slightly higher a_w_ value was recorded for GL-enriched pasta. However, the value did not exceed the acceptable limit (0.6) for any sample. Water activity is an important parameter that determines the shelf life of food products. Pasta with moisture content below 12% (a_w_ < 0.6) exhibits good microbiological resistance [24].

#### Color Parameters of Pasta

Several studies have investigated the enrichment of pasta with dried and powdered or pureed fruits, vegetables, or compounds extracted from these raw materials in recent years [6]. It has been found that additives of plant origin can significantly influence the color of WP [12]. Moreover, the color attributes of raw pasta have been proven to be excellent indicators of pasta cooking properties [25]. Table 2 presents the values of color coordinates determined for raw and cooked pasta samples.

* PL0, PL1, PL2, PL3, PL4, PL5, pasta with 0, 1, 2, 3, 4 and 5 g/100 g of green leek, respectively, L*—lightness, a*—redness, b*—yellowness, ΔE—total color difference, ** The values designated by the different small letters (a–e) in the columns of the table are significantly different (α = 0.05).

Semolina is the best raw material for WP. Due to the high content of carotenoids, WP flour appears light yellow in color [1,2]. In this study, due to lower values of lightness (L*), raw pasta samples enriched with GL were darker than semolina pasta (PL0). The lightness of GL-enriched raw pasta decreased from 68.07 (PL0) to 61.56 (PL5), whereas the value of the redness parameter (a*) decreased from 4.32 (PL0) to 2.58 (PL5). The highest a* value in relation to PL0 was observed in the PL3 sample, while the lowest value was recorded for the PL5 sample. The largest change in yellowness (b*) was also found in PL3, with a value of 22.25 for PL0 and 26.54 for PL3. The total color difference (ΔE) between control and enriched pasta samples increased from 2.22 (PL2) to 6.89 (PL5). Visible color changes were observed in the PL5 pasta samples enriched with the highest concentration of GL in comparison to PL0. A significant linear correlation was found between GL enrichment and ΔE (*r* = 0.95, *p* < 0.05). This effect was due to the darker color of the additive used. GL contains a high amount of chlorophyll pigments [26], which are mainly responsible for color changes in pasta samples.

Importantly, cooking enhanced the differences between the color of control and enriched pasta. The ΔE values of cooked GL-enriched pasta varied from 5.60 to 11.06, and a significant correlation was also found between GL enrichment and ΔE (*r* = 0.91, *p* < 0.05). In most of the studied pasta samples, cooking caused a significant increase in the L* value. The lightness of cooked pasta samples ranged from 67.12 for PL3 to 75.51 for PL0. Moreover, redness decreased in all pasta samples after cooking. The highest a* value was observed for PL2 (2.81), while the lowest was for PL0 (1.56). In the case of yellowness, cooked GL-enriched pasta samples were characterized by significantly higher b^*^ values compared to the control sample. The value of yellowness changed from 18.07 for PL0 to 23.84 for PL5. Other authors have also reported that cooking had a significant impact on pasta color, and cooked WP was lighter compared to raw pasta [19].

### 2.2. Cooking Properties of Pasta

The cooking properties of pasta are very important from the consumers’ point of view [27]. Studies have shown that GL addition caused an increase in the optimum cooking time (OCT) of WP from 9.03 min for PL0 to 11.63 min for PL5 (Table 3). OTC has been determined by the disappearance of the white core when the pasta strand is squeezed between a pair of glass plates. It is the time to cook the pasta to fully hydrate it.

A significant and positive correlation was observed between cooking time and GL addition (*r* = 0.97, *p* < 0.05). Moreover, GL caused a significant decrease in weight increase (WI) from 3.40 (PL0) to 2.34 and 2.37 (PL1 and PL5, respectively). Longer cooking time can increase the weight of pasta [27]. The lower WI index of pasta was observed with the increased addition of fruits [6]. For good-quality pasta, the appropriate cooked weight is about three times the dry weight [28]. This has been confirmed by Michalak-Majewska et al. [19] for pasta enriched with onion skin. The addition of other vegetables also caused a decrease in WI [29]. Furthermore, GL enrichment significantly increased material loss during cooking (from 6.42% for PL0 to 7.20% for PL5; *r* = 0.96, *p* < 0.05). Sissons et al. [28] reported that in good-quality pasta, cooking loss (CL) should not exceed 8%. Although CL increased with the addition of GL, it did not exceed the acceptable level. A similar trend was observed by Bouasla [30] for pasta enriched with parsley leaves. In addition, pasta prepared from common wheat flour enriched with kañawa flour showed increased CL [31]. It was also observed that not only did vegetable additives increase CL, but dried bananas also caused higher CL due to the weakening of gluten structure [7].

### 2.3. Texture Properties

**Figure 1:**
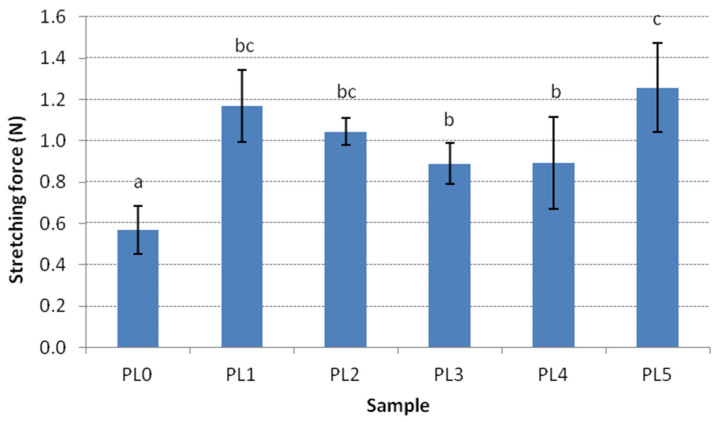
Changes in stretching force (N) with respect to GL enrichment. PL0, PL1, PL2, PL3, PL4, and PL5 denote pasta with 0, 1, 2, 3, 4, and 5 g/100 g of green leek, respectively. Values followed by the same letter (a–c) are not significantly different (*p* < 0.05), *n* = 5.

The study showed that the addition of GL caused a significant increase in the stretching force of cooked pasta (Figure 1). The highest stretching force was recorded for PL5 (1.26 N), and the lowest was recorded for PL0 (0.57 N). The stretching force was the highest for pasta with the longest OCT. A similar trend has been observed for WP enriched with cereal coffee. The lowest stretching force values were found for pasta with the lowest cooking time [32]. However, the cited study demonstrated that vegetable additives caused deterioration of the texture characteristics of pasta, which were determined based on cutting and stretching forces.

The addition of different compounds into wheat dough results in structural changes in the gluten network. These changes are often caused by dehydration of the gluten network during dough preparation [33]. A similar effect was observed in our study. The water absorption index of pasta significantly decreased during cooking. This suggests that enriched pasta absorbed less water than the control product. Water content has a strong impact on the mechanical properties of pasta [30]. The lesser the water content, the higher the strength of the pasta. Water decreases the glass transition temperature in the food matrix and is the most effective plasticizer in the food matrix, especially for hydrophilic food components. Usually, a softening effect in food is observed with an increased water concentration [34]. The key properties of water significant for its plasticizing effect are the capability of strong interactions with other polar molecules via hydrogen bonding and its high dielectric constant. The plasticizing activity is based on weakening hydrogen bonds and dipole–dipole intra- and inter-macromolecular interactions as a result of the shielding of attractive forces by water molecules [35]. This could explain the higher stretching forces observed for GL-enriched pasta compared to the control sample. Other authors also observed a similar relationship between the WI index and the stretching forces of pasta during a uniaxial extension test [36].

### 2.4. Total Phenolic Content and Antioxidant Activity of Cooked Pasta

The antioxidant activity (AA) of food products is an important parameter that provides information on a variety of factors, such as resistance to oxidation, the quantitative contribution of antioxidant substances, or AA that may occur inside an organism when the product is ingested [37]. Cooking affects the nutritional value of pasta enriched with vegetables because bioactive compounds present in the vegetables can penetrate into boiling water or undergo thermal degradation [27]. Table 4 presents the values of total phenolic content (TPC) and AA (determined using ABTS (2,2′-azinobis (3-ethylbenzothiazoline-6-sulfonate) and DPPH (2,2-diphenyl-1-picrylhydrazyl) assays) of cooked pasta.

GL caused a significant increase in phenolic content in pasta. The TPC of PL0 was 0.66 mg gallic acid equivalent (GAE)/g dry matter (d.m.), while the value was found to be more than twofold higher for PL5 (1.48 mg GAE/g d.m). A significant correlation was found between GL and TPC (*r* = 0.95, *p* < 0.05). GL also caused an increase in the AA of GL-enriched pasta compared to the control sample. However, significant differences in AA were found from control pasta when GL was added at concentrations of 3 and 4 g/100 g for ABTS and DPPH assays, respectively. As the amount of GL in the pasta recipe increased, the AA of cooked pasta also increased (half the maximum effective concentration or EC_50_ value decreased). In the case of ABTS, the EC_50_ value decreased significantly with the addition of GL from 22.20 mg d.m./mL for the control sample to 18.12 mg d.m./mL for PL5. A similar tendency was also observed for DPPH, and GL addition increased the ability of pasta to neutralize DPPH free radicals (EC_50_ value decreased). The EC_50_ value for DPPH was determined at 57.87 mg d.m./mL and 39.55 mg d.m./mL for PL0 and PL5, respectively. The correlation between GL enrichment and AA was statistically significant (*r* = 0.94 and 0.92 for ABTS and DPPH, respectively). The increase in AA may be caused by the bioactive compounds present in GL. A previous study showed that GL is a valuable source of biologically active compounds, especially phenolic acids, which are strong antioxidants [19]. The green part of leek also contains phenolic derivatives, with acylated kaempferol glycoside found to be dominant [20]. The results of the present study also indicated a significant correlation between TPC and ABTS radical scavenging activity (*r* = 0.98, *p* < 0.05) and TPC and DPPH radical scavenging activity (*r* = 0.97, *p* < 0.05). Similar dependencies were reported by other authors for pasta enriched with onion skin [19], parsley leaf [30], and cereal coffee [32]. This suggests that the powder from the green parts of the leek can be successfully used in nutritionally valuable pasta recipes.

### 2.5. Sensory Properties

**Figure 2:**
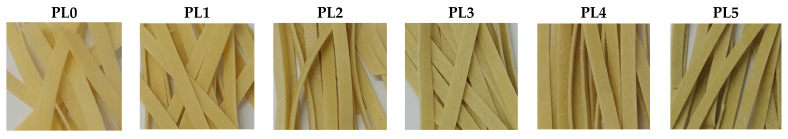
Pasta enriched with GL. PL0, PL1, PL2, PL3, PL4, and PL5 denote pasta with 0, 1, 2, 3, 4, and 5 g/100 g of green leek, respectively.

Pasta samples enriched with 0–5 g/100 g of GL are shown in Figure 2. The addition of vegetable additives usually has a negative impact on the strength of the gluten network, causing undesirable changes in the sensory characteristics of pasta [27]. Therefore, the amount of additive should be optimized to maintain the desired sensory characteristics of the final product.

The addition of GL up to 2 g/100 g did not have any negative effect on the tested sensory attributes (Table 5). In terms of smell, PL0 was rated the best (6.90), as it had a distinct pleasant aroma, whereas PL2 was rated a little worse, with a slightly perceptible smell of leek (6.70). In terms of taste, PL1 and PL2 were rated higher (6.80 and 6.60, respectively). Pasta enriched with GL had a much noticeable leek flavor. The addition of GL also affected the color of pasta. The color of enriched pasta varied from gray to slightly green, but it was solid. PL1 samples were rated to have the best color (6.80), whereas PL4 was judged to have the worst color (3.90). Enrichment with a higher concentration of GL (>3 g/100 g) caused a decline in all tested sensory attributes. GL also had a negative effect on the adhesiveness of pasta. PL5 was rated the worst for adhesiveness (4.30). Adhesiveness characterizes the stickiness of pasta during consumption. The increased leek-pasta stickiness is probably caused by the higher cooking loss of enriched product, related to the amount of amylose leached out from the gelatinized starch granules during cooking [38]. To summarise, PL0 and PL2 samples received the highest score for the overall quality (both 6.70). PL1 received a slightly lower score for overall acceptability (6.60). PL5 obtained a worse score for taste and color and had reduced firmness. Sensory evaluation revealed that pasta prepared with GL (up to 3 g/100 g) had good overall acceptability. Some authors investigated the effect of a leek supplement on the sensory characteristics of bread and cheese and, based on the sensory analysis, reported that wheat flour could be fortified with up to 1 g/100 g of leek powder without any adverse effect on consumer acceptability [20]. On the other hand, cheese enriched with *A. ampeloprasum* showed a change in taste in the sensory analysis, which indicated that the addition of *Allium* vegetables as a plant additive could increase the sensory properties of the final product [39].

## 3. Materials and Methods

### 3.1. Raw Materials and Chemicals

Semolina (granulation in the range of 200–300 μm, d.m. 84.5%, ash 0.95%, protein 12.0%, fat 1.2%, and fiber 3.9%) was purchased from Batom.pl (Poland, Cracow). Leeks (*A. porrum*, cv. Bartek) were collected from Lublin (Poland) region in the autumn of 2020. For the preparation of enriched pasta, the green part of leeks (d.m. 88.4%, ash 5.1%, protein 12.3%, fat 2.3%, fiber 8.2%) was powdered following the methodology of Biernacka et al. [20]. Briefly, the collected leeks were thoroughly cleaned and stripped of the root shaft, and white parts were separated from the green parts of the leek. Then, the green parts were cut into slices (discs with about 0.5 cm thickness) and freeze-dried. The process was performed at 20 °C and at a constant pressure value of 100 Pa using a freeze-dryer (ALPHA 1–4, Martin Christ Gefriertrocknungsanlagen GmbH, Osterode am Harz, Germany). After drying, the leek samples (about 3% of moisture content) were powdered using a knife mill (GRINDOMIX GM-200, Retsch, Haan, Germany; 1000 W, 10,000 rpm). The particles below 300 μm were used for future studies and pasta preparation.

All chemicals used in the study (Folin–Ciocalteu reagent, DPPH, ABTS) were of analytical grade. Other chemicals were purchased from Sigma Aldrich (St. Louis, MO, USA).

### 3.2. Preparation of Pasta

The pasta was prepared with semolina, which was partially replaced with GL at concentrations of 0, 1, 2, 3, 4, and 5 g/100 g. A KitchenAid Heavy Duty mixer (model T-5KPM5EER, Greenville, SC, USA) was used to prepare the dough and pasta. The pasta was prepared from semolina, tap water (an amount of about 40% moisture, 140 g of water was used for 350 g of flour), and GL. All ingredients were mixed for 5 min at 150 rpm. A flat stirrer (KitchenAid 5KSM5TH3PSS, Greenville, SC, USA) was used to knead the dough. The dough was laminated into sheets up to 150 mm in width and 2.0 mm thick by using a pasta rolling device (5KSMPSA, Greenville, SC, USA). The strands of pasta (1.5 mm thick) were obtained with a special attachment (5KSMPRA, Greenville, SC, USA). The obtained product was dried on a pasta dryer (KitchenAid 5KPDR, Greenville, SC, USA) for 24 h at 25 °C and 30% relative humidity in a climate chamber (ICH 256, Düsseldorf, Germany) until the moisture of the pasta reached between 11.5% and 12% (wet basis). Pasta samples with a length of about 250 mm, a thickness of about 1, 5 mm, and width of 5 mm were obtained.

### 3.3. Evaluation of Chemical and Physical Properties of Pasta

#### 3.3.1. Determination of the Basic Chemical Composition

GL, uncooked control pasta (PL0), and pasta with GL were analyzed to determine the content of d.m., crude protein, crude ash, and crude fat using Method 44-15A, Method 08-01, Method 46-06, and Method 30-10, respectively. The analysis was performed in triplicate and in accordance with the AOAC standards [40].

#### 3.3.2. Measurement of Water Activity

Water activity (a_w_) was measured in pasta at 20 °C using a LabMaster (Novasina AG, Lachen, Switzerland) as described by Serin et al. [41].

#### 3.3.3. Evaluation of Color Coordinates

The color coordinates of uncooked and cooked control pasta (PL0) and pasta with GL were determined in the CIEL*a*b* system using a colorimeter (CR-400C Chroma Metre 115, Minolta, Colour Lab, Osaka, Japan). The CIEL*a*b* system includes L*, a*, and b* parameters. L* denotes lightness, which ranges from 0 (perfect black body) to 100 (perfect white body). a* specifies the change of green color (−a*) to red color (a*), while b * specifies the change of color from blue (−b*) to yellow (b*) [42]. The change in color due to GL fortification was determined by calculating the color differential index (ΔE) as described by Monteiro et al. [43].

### 3.4. Cooking Quality of Pasta

#### 3.4.1. Optimal Cooking Time

OCT was determined in accordance with AACC 66-50 [44].

#### 3.4.2. Weight Increase Index and Cooking Loss

WI index and CL were calculated as described by Bonomi et al. [45] and Biernacka et al. [11], respectively.

### 3.5. Texture Analysis

Texture analysis was performed on a Zwick testing machine (Zwick GmbH & Co., Ulm, Germany). Cooked pasta (Section 3.4.1) was subjected to a stretching test as described by Wójtowicz [46]. Pasta elongation was tested on Tensile Kiefer Dough and Gluten Extensibility Rig attached to Instron 5564 with a 50 N head (Stable Micro Systems Ltd., Godalming, UK). The tensile test speed was set at 3.3 mm⋅s^–1^. A single sample of cooked pasta was placed under a plastic cover on a test table for testing. During the tensile test, the stretching force was assessed using a computer program. The values shown in the curves are the average of five replicates.

### 3.6. Total Phenolic Content and Antioxidant Activity

For preparing the extracts, the ground dried sample was extracted with 50% methanol (shaken thrice for 30 min). The resulting homogenate was centrifuged at 4000 rpm for 10 min at 4 °C [47]. Extraction was performed twice. The obtained supernatants were pooled and used for further analyses.

TPC was determined by the Folin–Ciocalteu method [48], and the result was expressed as GAE/g d.m.

AA of the extracts was determined by analyzing:

- The ability to scavenge ABTS free radicals using the method of Re et al. [49]; and
- The ability to neutralize DPPH free radicals, as described by Brand-Williams et al. [50].

The results of the AA analysis were expressed as the EC_50_ index.

The potency of a compound is expressed as EC_50_, which refers to the concentration that induces a response halfway between the baseline and the maximum. The strength of a compound is inversely related to its EC_50_ value, and the lowest EC_50_ is associated with the strongest effect [51].

### 3.7. Sensory Evaluation

Cooked pasta samples were evaluated for smell, color, taste, and consistency. These features were evaluated in terms of firmness and adhesiveness in an overall assessment. The evaluation panel consisted of 37 untrained Polish consumers (24–45 years old; 23 women and 14 men). The hedonic scale used for measuring the examined features had scores ranging from 1 (least acceptable) to 7 (most acceptable). When assessing organoleptic characteristics, adequate lighting was ensured, and the room was made free of foreign odors [45].

### 3.8. Statistical Analysis

Experimental data were subjected to a one-way analysis of variance using STATISTICA 6 (StatSoft, Inc., Tulsa, OK, USA). The significance of differences between the means was determined using Tukey’s test. All measurements were performed in triplicate, except for the texture analysis. The stretching test was performed in five replications. Pearson’s correlation coefficient was also determined. All tests were performed at a significance level of α = 0.05.

## 4. Conclusions

Enrichment of pasta with GL resulted in a slight but significant decrease in protein content but an increase in ash, fiber, and fat content. However, GL affected the color of cooked pasta by decreasing the lightness and increasing the yellowness of this product. GL also influenced the cooking properties of pasta, and it increased the OCT and CL and decreased the WI. Furthermore, the addition of GL caused a significant increase in the stretching force of cooked pasta. GL also significantly enhanced the antioxidant properties of pasta. On the other hand, an increased amount of GL in the pasta recipe led to an increase in adhesiveness of the pasta and had a negative effect on the smell, taste, and color of the product. To sum up, our study confirmed that GL (up to 3 g/100 g) can be a useful additive to semolina flour in the production of pasta with enhanced nutritional and pro-health value as well as acceptable sensory properties.

## Figures and Tables

**Table 1:** Chemical composition (%) and water activity of the pasta enriched with green leek.

Sample	Parameter					
	Dry Matter (%)	Protein (%)	Fiber (%)	Ash (%)	Fat (%)	Water Activity
PL0 *	88.27 ± 0.03 ^a^**	15.62 ± 0.06 ^d^	4.05 ± 0.03 ^a^	0.97 ± 0.02 ^a^	0.25 ± 0.02 ^a^	0.537 ± 0.003 ^a^
PL1	88.65 ± 0.03 ^b^	15.25 ± 0.02 ^b^	4.08 ± 0.02 ^a^	1.02 ± 0.02 ^b^	0.30 ± 0.03 ^ab^	0.561 ± 0.003 ^bc^
PL2	88.79 ± 0.02 ^d^	15.23 ± 0.02 ^b^	4.10 ± 0.07 ^ab^	1.10 ± 0.01 ^c^	0.30 ± 0.01 ^ab^	0.563 ± 0.005 ^c^
PL3	88.76 ± 0.04 ^cd^	15.25 ± 0.02 ^b^	4.12 ± 0.04 ^bc^	1.21 ± 0.02 ^d^	0.32 ± 0.02 ^bc^	0.551 ± 0.007 ^b^
PL4	88.70 ± 0.05 ^bcd^	15.14 ± 0.01 ^a^	4.22 ± 0.06 ^cd^	1.27 ± 0.02 ^e^	0.36 ± 0.02 ^cd^	0.570 ± 0.004 ^c^
PL5	88.67 ± 0.03 ^bc^	15.09 ± 0.02 ^a^	4.28 ± 0.08 ^d^	1.19 ± 0.01 ^d^	0.39 ± 0.01 ^d^	0.560 ± 0.004 ^bc^

* PL0, PL1, PL2, PL3, PL4, PL5, pasta with 0, 1, 2, 3, 4 and 5 g/100 g of green leek, respectively. ** The values designated by the different small letters (a–e) in the columns of the table are significantly different (α = 0.05).

**Table 2:** The color parameters of uncooked and cooked pasta.

Sample	Colour Parameters			
	L*	a*	b*	ΔE
**Uncooked Pasta**				
PL0 *	68.07 ± 1.16 ^c^**	4.32 ± 0.43 ^d^	22.25 ± 1.47 ^ab^	-
PL1	68.11 ± 0.77 ^c^	3.30 ± 0.09 ^ab^	24.58 ± 0.55 ^bc^	2.54 ± 0.94 ^a^
PL2	66.21 ± 0.74 ^c^	4.13 ± 0.14 ^cd^	23.45 ± 0.82 ^ab^	2.22 ± 0.34 ^a^
PL3	65.77 ± 1.20 ^bc^	5.18 ± 0.50 ^e^	26.54 ± 1.92 ^c^	4.94 ± 3.32 ^b^
PL4	62.77 ± 1.31 ^ab^	3.48 ± 0.08 ^bc^	20.61 ± 0.96 ^a^	5.61 ± 0.86 ^bc^
PL5	61.56 ± 1.76 ^a^	2.58 ± 0.20 ^a^	23.68 ± 1.24 ^abc^	6.89 ± 1.01 ^c^
**Cooked Pasta**				
PL0	75.51 ± 1.36 ^c^	1.56 ± 0.29 ^a^	18.07 ± 0.20 ^a^	-
PL1	72.32 ± 1.92 ^bc^	2.73 ± 0.03 ^b^	22.20 ± 0.91 ^b^	5.60 ± 1.90 ^a^
PL2	68.87 ± 2.05 ^ab^	2.81 ± 0.13 ^b^	23.80 ± 0.86 ^bc^	9.14 ± 1.82 ^b^
PL3	67.12 ± 2.59 ^a^	2.33 ± 0.15 ^b^	22.12 ± 0.75 ^b^	9.40 ± 2.11 ^b^
PL4	67.33 ± 1.28 ^a^	2.54 ± 0.32 ^b^	23.84 ± 0.88 ^bc^	10.10 ± 2.13 ^bc^
PL5	67.29 ± 0.81 ^a^	1.75 ± 0.16 ^a^	25.43 ± 0.79 ^c^	11.06 ± 1.27 ^c^

* PL0, PL1, PL2, PL3, PL4, PL5, pasta with 0, 1, 2, 3, 4 and 5 g/100 g of green leek, respectively, L*—lightness, a*—redness, b*—yellowness, ΔE—total color difference, ** The values designated by the different small letters (a–e) in the columns of the table are significantly different (α = 0.05).

**Table 3:** Cooking properties of pasta.

Sample	Parameters		
	OCT (min)	WI (kg CP/kg UP)	CL (g/100 g of Pasta)
PL0 *	9.03 ± 0.23 ^a^**	3.40 ± 0.02 ^e^	6.42 ± 0.03 ^a^
PL1	9.50 ± 0.20 ^ab^	2.34 ± 0.01 ^a^	6.49 ± 0.03 ^b^
PL2	9.90 ± 0.26 ^b^	2.46 ± 0.03 ^cd^	6.58 ± 0.02 ^c^
PL3	11.20 ± 0.30 ^c^	2.52 ± 0.03 ^d^	6.75 ± 0.02 ^d^
PL4	11.43 ± 0.21 ^c^	2.43 ± 0.02 ^bc^	7.17 ± 0.02 ^e^
PL5	11.63 ± 0.35 ^c^	2.37 ± 0.02 ^ab^	7.20 ± 0.01 ^e^

* PL0, PL1, PL2, PL3, PL4, PL5, pasta with 0, 1, 2, 3, 4 and 5 g/100 g of green leek, respectively. OCT—optimum cooking time (min), WI—weight increase index (kg * kg^−1^), CL—cooking loss (kg * kg^−1^), CP—cooked pasta, UP—uncooked pasta, ** Mean ± SD, *n* = 3. Values followed by the same letter (a–e) in the same rows are not significantly different (*p* < 0.05).

**Table 4:** Total phenolic content (TPC) and antioxidant activity (AA) of cooked pasta.

Sample	Parameters		
	TPC (mg GEA/g d. m.)	ABTS EC_50_ (mg d. m./mL)	DPPH EC_50_ (mg d. m./mL)
PL0 *	0.66 ± 0.02 ^a^**	22.20 ± 0.44 ^d^	60.63 ± 7.29 ^c^
PL1	0.77 ± 0.04 ^ab^	22.11 ± 0.10 ^d^	57.87 ± 1.25 ^bc^
PL2	0.91 ± 0.02 ^bc^	21.72 ± 0.18 ^d^	54.89 ± 5.40 ^bc^
PL3	0.96 ± 0.01 ^bc^	20.99 ± 0.12 ^c^	51.60 ± 0.44 ^bc^
PL4	1.09 ± 0.04 ^c^	19.77 ± 0.21 ^b^	49.11 ± 1.90 ^ab^
PL5	1.48 ± 0.16 ^d^	18.12 ± 0.07 ^a^	39.55 ± 3.72 ^a^

* PL0, PL1, PL2, PL3, PL4, PL5, ** pasta with 0, 1, 2, 3, 4 and 5 g/100 g of green leek, respectively. ** Mean ± SD, *n* = 3, separate statistical analyses of TPC, ABTS and DPPH were performed, different small letters (a–d) in the columns of the table mean significant differences between means (*p* < 0.05).

**Table 5:** Sensory characteristics of cooked pasta fortified green leek powder.

Sample	Sensory Attribute					
	Smell	Taste	Colour	Firmness	Adhesiveness	Overall
PL0 *	6.90 ± 0.32 ^c^**	6.10 ± 0.57 ^bc^	6.70 ± 0.48 ^c^	6.40 ± 0.70 ^bc^	6.60 ± 0.52 ^c^	6.70 ± 0.48 ^b^
PL1	6.40 ± 0.97 ^bc^	6.80 ± 0.42 ^c^	6.80 ± 0.42 ^c^	6.50 ± 0.53 ^c^	6.50 ± 0.53 ^c^	6.60 ± 0.52 ^b^
PL2	6.70 ± 0.48 ^bc^	6.60 ± 0.70 ^c^	6.10 ± 0.88 ^bc^	6.30 ± 0.48 ^bc^	6.10 ± 0.57 ^bc^	6.70 ± 0.48 ^b^
PL3	5.50 ± 1.27 ^ab^	5.20 ± 0.63 ^b^	4.80 ± 1.14 ^ab^	5.10 ± 0.74 ^ab^	5.20 ± 0.92 ^ab^	5.10 ± 0.74 ^a^
PL4	4.90 ± 0.88 ^a^	5.00 ± 0.82 ^ab^	3.90 ± 1.10 ^a^	4.40 ± 0.97 ^a^	4.70 ± 0.67 ^a^	4.50 ± 0.97 ^a^
PL5	4.60 ± 1.51 ^a^	3.90 ± 1.66 ^a^	4.00 ± 1.83 ^a^	4.10 ± 1.91 ^a^	4.30 ± 1.57 ^a^	4.00 ± 1.83 ^a^

* PL0, PL1, PL2, PL3, PL4, PL5, pasta with 0, 1, 2, 3, 4 and 5 g/100 g of green leek, respectively. Acceptability on a 7-point hedonic scale. ** Mean ± SD, *n* = 3, values followed by the same letter (a–c) in the same rows are not significantly different (*p* < 0.05).

## Data Availability

The data presented in this study are available on request from the corresponding author.

## Sample Availability

Samples of the compounds are available from the authors.
